# Multi trait stability indexing and trait correlation from a dataset of sweet potato (*Ipomoea batatas* L.)

**DOI:** 10.1016/j.dib.2023.109995

**Published:** 2023-12-21

**Authors:** Zakaria Alam, Sanjida Akter, Md. Anwar Hossain Khan, Md. Harunor Rashid, Md. Iqbal Hossain, Abul Bashar, Umakanta Sarker

**Affiliations:** a Tuber Crops Research Centre, Bangladesh Agricultural Research Institute, Gazipur-1701, Bangladesh; b Entomology Division, Bangladesh Rice Research Institute, Gazipur-1701, Bangladesh; c Sher-e-Bangla Agriculture University, Dhaka 1213, Bangladesh; d Department of Genetics and Plant Breeding, Faculty of Agriculture, Bangabandhu Sheikh Mujibur Rahman Agricultural University, Gazipur-1706, Bangladesh

**Keywords:** MTSI, Sweet potato traits, Factor analysis, Broad sense heritability, Selection gain

## Abstract

A study was conducted in five regions of Bangladesh, specifically Gazipur, Bogura, Jamalpur, Jashore, and Chattogram, each characterized by suitable agro-ecologies for sweet potato cultivation. The purpose of this data article was to demonstrate the correlations between traits and the selection of stable varieties based on the multi-trait stability index (MTSI). The data indicated a direct link between multiple characteristics and both the yield and factors contributing to yield. This implies that enhancing these traits might result in a higher overall production of sweet potato storage roots. Furthermore, the factor analysis for MTSI demonstrated that the desired goal for selection was achieved for all traits, except for mean vine length (VL) and storage root dry weight (DW). The broad sense heritability ranged from 0 to 0.97, and the selection gain percentage ranged from 0 to 42.8. The MTSI analysis identified the sweet potato variety BARI Mistialu-15 as the most stable among the other studied varieties.

Specifications Table

**Table:** 

Subject	Agricultural and Biological Science
Specific subject area	Agronomy and Crop Science
Data format	Raw
Type of data	Table and Figures
How the data were collected	Data were acquired through a study carried out under field conditions involving five distinct sweet potato varieties. Measurements were taken using a measuring scale, slide callipers, and a weight machine to gather the agronomic data. Information pertaining to dry weight of roots was obtained through the utilization of a Laboratory oven. The ‘metan’ package of the R software facilitated the derivation of figures and tables representing the data by calculating eight agronomic traits and one quality trait.
Data source location	Five districts in Bangladesh: Gazipur, Bogura, Jamalpur, Chattogram, and Jashore. The study area lies within Bangladesh's geographic coordinates, ranging from 23.6850° N latitude to 90.3563° E longitude. It spans elevations from 10 m (Coastal South) to 105 m (North) above sea level.
Data accessibility	https://data.mendeley.com/datasets/v896jzf9s5/1
Related research article	Z. Alam, S. Akter, M.A.H. Khan, M.S. Alam, S. Sultana, S. Akhter, M.M. Rahman, M.M. Islam, 2023. Yield performance and trait correlation of BARI released sweet potato varieties studied under several districts of Bangladesh. Heliyon. 9, e18203. DOI:https://doi.org/10.1016/j.heliyon.2023.e18203.

## 1 Value of the Data

• This dataset presents information on the relationship between sweet potato traits and their impact on storage root yield.
• The dataset in this article supplies data to researchers, farmers, and industry users on the multi trait stability of sweet potato genotypes grown in different locations of Bangladesh.
• The provided data has a potential for assisting in crop improvement programs and genetic studies, particularly in analyzing the stability of sweet potato based on specific traits of importance.
• The dataset demonstrates the usefulness of the MTSI index in speeding up the selection of stable genotypes in crop breeding programs.

## 2 Background

In the context of Bangladesh, there exists a pressing requirement to ascertain sweet potato genotypes that exhibit the high yield potential and demonstrate stability in varying environmental conditions. The original article highlights the yield potential and correlation of traits of the examined varieties. Nevertheless, the incorporation of the Multi Trait Stability Index (MTSI) in the dataset will introduce a novel aspect, encompassing stability ranking of studied varieties based on a multi-dimensional trait index.

## 3 Data Description

The challenge of selecting genotypes for breeders is posed by the need to adapt to various environmental conditions, as the specific environment influences and interconnects the phenotypic traits of plants. The concept of a plant ideotype encompasses a set of desirable traits that can be independently measured in the field. By utilizing this approach, the selection process becomes more streamlined, systematic, and dynamic, facilitating a better understanding of the genotype-environment interaction in multi-environment trials [1]. This study conducted in Bangladesh involved the cultivation of five genotypes of sweet potato varieties across five different locations. The dataset presented in this article comprises two figures and one table.

**Fig. 1:**
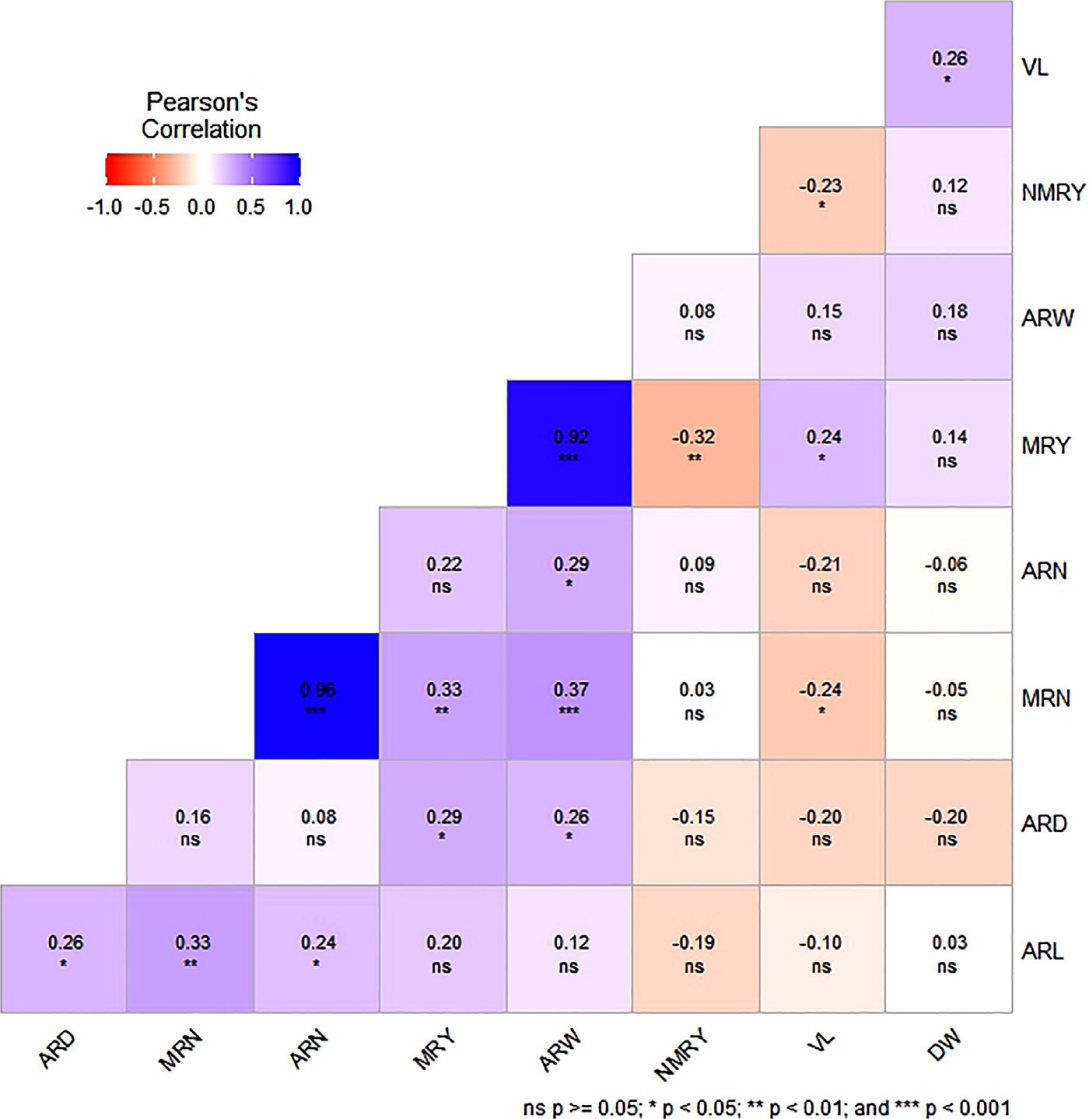
A correlation matrix using Pearson coefficients, visually represented as a heat map, displays the relationships between various agronomic factors, quality traits, and the yield of marketable storage roots in different varieties of sweet potatoes cultivated across five locations in Bangladesh.

Fig. 1 presents the correlation coefficients utilized to evaluate the associations among eight yield-contributing traits and one quality trait, employing data from five different locations and five distinct varieties. The findings demonstrated noteworthy positive correlations (*p* ≤ 0.05) among all the examined traits, with the exception of non-marketable root yield. Consequently, the acquired data from the correlation analysis disclosed that positively correlated traits serve as pivotal elements that impact the root yield in sweet potatoes.

Table 1 displays the results of the factor analysis, which reveal the broad sense heritability, selection gain percentage, desired selection sense, and the achievement status of the goal based on factor analysis of selected sweet potato genotypes using nine traits. The factor analysis revealed that the eigenvalues of three factors were greater than 1, leading to the inclusion of these three factors in Table 1. These factors formed three distinct groups consisting of nine traits under study. The desired selection sense was to increase for all traits, with the exception of non-marketable storage root yield (NMRY). The goal was successfully achieved for all traits, with the exception of mean vine length (VL) and the dry weight of storage root (DW). Among the studied traits, the broad sense heritability was highest for DW (0.97), followed by average root diameter (ARD) (0.93), average root length (ARL) (0.88), NMRY (0.87), VL (0.54), and marketable root yield (MRY) (0.41). Furthermore, the selection gain percentage for these traits was also higher, indicating that the selected genotype shows great promise in terms of the heritability of a few agronomic traits. The selected genotype, however, possesses a significant weakness in DW.

**Table 1:** Factor analysis for selection gain (%), broad-sense heritability, initial selection sense and selection goal status for selected variety using nine sweet potato traits achieved through the indexing of MTSI.

Variables	Factor	Broad sense heritability	Selection gain (%)	Sense	Goal
MRN	FA1	0.344	2.17	increase	100
MRY		0.415	4.58	increase	100
ARN		0.142	0.336	increase	100
ARW		0	0	increase	100
ARD	FA2	0.934	15.3	increase	100
NMRY		0.872	−42.8	decrease	100
VL	FA3	0.541	−1.78	increase	0
ARL		0.878	11.3	increase	100
DW		0.967	−7.88	increase	0

VL, Mean vine length (cm); ARL, storage root length (cm) ; ARD, storage root diameter (cm) ARN, average storage roots per plant; ARW, average storage roots weight per plant (kg) MRN, mmarketable storage root number per plant; MRY, marketable storage root yield (t/ha); NMRW, non-marketable storage root yield (t/ha); DW, dry matter content (%).

**Fig. 2:**
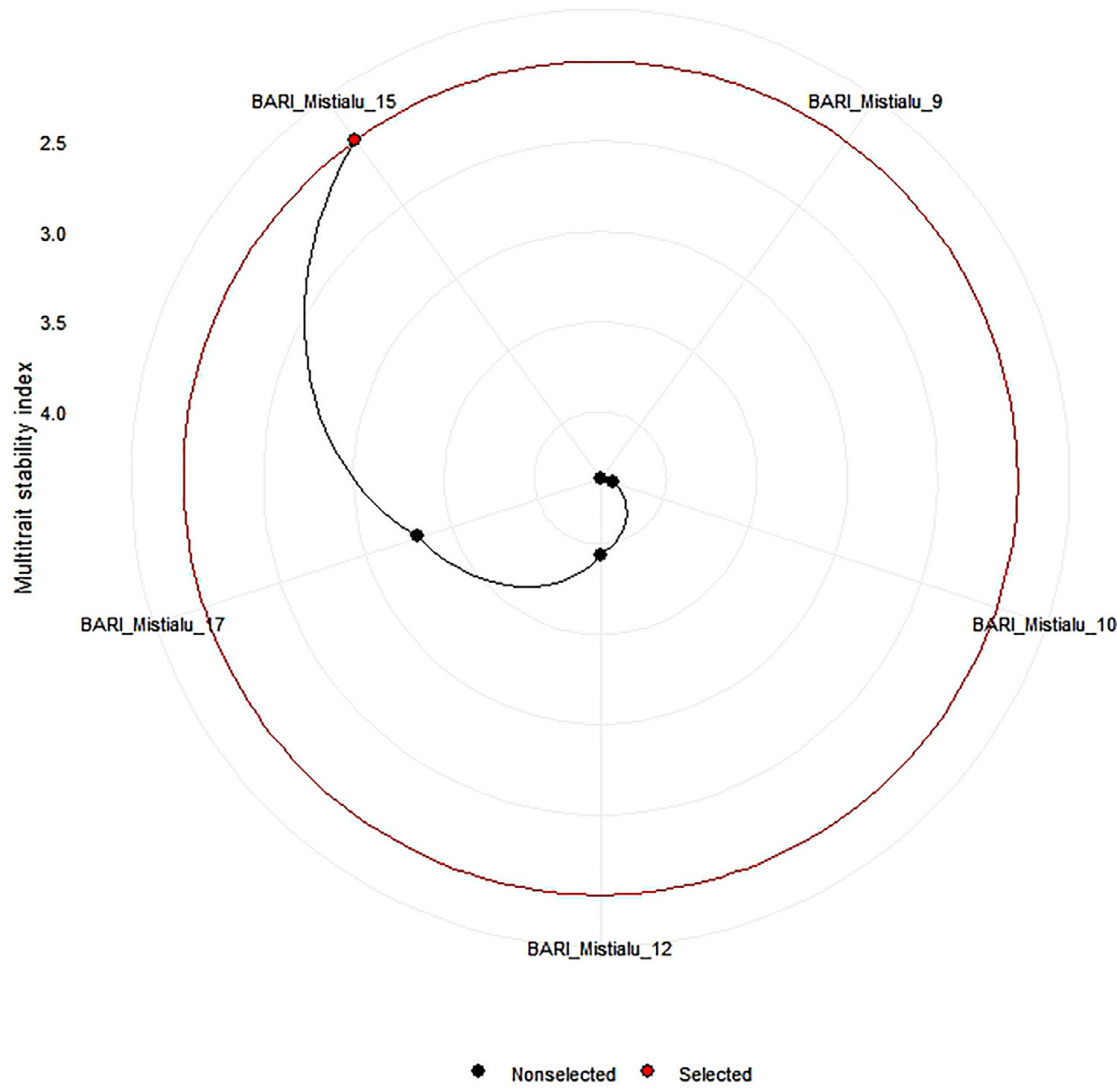
Ranking of stable sweet potato varieties based on MTSI index achieved through utilization of specific selection sense.

Fig. 2 presents the representation of the order of the most stable genotype determined by the multi-trait stability indexing (MTSI). Within this context, the red circle symbolizes the threshold for selecting the stable genotype [2]. The sweet potato genotype called BARI Mistialu-15 stands out as the most stable compared to other genotypes.

## 4 Experimental Design, Materials and Methods

### 4.1 Description of the study area

The study took place in the 2021–22 growing season across five districts in Bangladesh: Gazipur, Bogura, Jamalpur, Chattogram, and Jashore. The research sites are located in Bangladesh, spanning latitudes 23.6850° N and longitudes 90.3563° E, covering elevations ranging from 10 m (Coastal South) to 105 m (North) above sea level.

### 4.2 Experimental design and materials

Five renowned varieties of sweet potatoes, namely BARI Mistialu-9, BARI Mistialu-10, BARI Mistialu-12, BARI Mistialu-15, and BARI Mistialu-17, were utilized for the conducted experiment at the TCRC, BARI, Gazipur. Detailed information regarding these varieties can be found in the supplementary Table 1. The study employed a randomized complete block design (RCBD) with three replications.

### 4.3 Experimental techniques

The management practices for sweet potato were done following a standard procedure of Alam et al. [3]. The harvesting took place after 130 days after vine planting, wherein each genotype consisted of 10 plants. In order to conduct a thorough quality analysis, the laboratory of Postharvest Technology Division at BARI collected and examined the storage roots of each genotype, each weighing 500 g. The following data was gathered in accordance with the methodology outlined by Alam et al. [3]: mean vine length (cm) (VL), storage root length (cm) (ARL), storage root diameter (cm) (ARD), average storage roots per plant (ARN), average storage roots weight per plant (kg) (ARW), marketable storage root number per plant (MRN), marketable storage root yield (t/ha) (MRY), non-marketable storage root yield (t/ha) (NMRW) and dry matter content (%) (DW).

### 4.4 Statistical analysis

Correlation coefficient of nine sweet potato traits was analysed using ‘metan’ package of R software. MTSI was computed to calculate the multi trait stability index using following formula [4].

$$\text{MTS}I_{i}={\left[\sum_{j=1}^{f}\left(\gamma_{ij}-\gamma_{j}\right)\right]}^{0.5}$$

The stability index for the *i*th genotype, labeled as MTSI_i_, is determined based on multiple traits. The score of the *i*th genotype in the *j*th factor is represented as $\gamma_{ij}$, while the score of the ideal genotype in the same factor is denoted as $\gamma_{j}$. Calculations for genotype and trait scores were conducted through factor analysis.

The length of nine variables' vector, where these variables were utilized for ideotype planning. Among nine variables, all the variable were planned with a higher desired sense except for NMRY, which was set for a low sense in analysing the MTSI index.

## Limitations

The dataset only covers one growing season so it may not capture the variability across different years. Factors like climate, soil, and pests can affect sweet potato yield and change every year.

## Ethics Statement

All authors have read and follow the ethical requirements for publication in Data in Brief and our work meets these requirements. Our work does not involve studies with animals and humans.

## CRediT authorship contribution statement

**Zakaria Alam:** Conceptualization, Methodology, Software, Writing – original draft. **Sanjida Akter:** Data curation, Software. **Md. Anwar Hossain Khan:** Visualization, Investigation, Supervision. **Md. Harunor Rashid:** Investigation, Supervision. **Md. Iqbal Hossain:** Software, Validation. **Abul Bashar:** Software. **Umakanta Sarker:** Writing – review & editing.

## Data Availability

Multi trait stability indexing and trait correlation from a dataset of sweet potato (Ipomoea batatas L.) (Original data) (Mendeley Data)

## Appendix Supplementary materials

Image, application 1
