# Supplementary material for: Multi trait stability indexing and trait correlation from a dataset of sweet potato (Ipomoea batatas L.)
Source: Data Brief. 2023 Dec 21;52:109995. doi: 10.1016/j.dib.2023.109995 (PMC10788196; doi:10.1016/j.dib.2023.109995)
Supplement: Supplementary file 1 [file mmc1.docx]

Supplementary table 1. Descriptions of five BARI released sweet potato varieties

| Genotype | Origin | **Altitude (m)** | **Year of release** | **Stem color** | **Skin Color** | **Maturity days** | **Flesh color** | **Potential Yield (t/ha)** |
| --- | --- | --- | --- | --- | --- | --- | --- | --- |
| BARI Mistialu-9 | Peru | 1555 | 2008 | Green | Red | 120-130 | Orange | 25-30 |
| BARI Mistialu-10 | Bangladesh | 10-105 | 2013 | Green | Brown | 120-130 | Cream | 30-35 |
| BARI Mistialu-12 | Peru | 1555 | 2013 | Green | Off white | 120-130 | Cream | 35-40 |
| BARI Mistialu-15 | Peru | 1555 | 2017 | Green | Pink | 120-130 | Orange | 35-40 |
| BARI Mistialu-17 | Indonesia | 367 | 2021 | Green | Purple | 120-130 | Purple | 30-35 |
